# Long-term changes in chemical components in the meadow pipit (*Anthus pratensis*) in the formerly heavily polluted Eastern Sudetes Mountains

**DOI:** 10.1007/s11356-025-36446-9

**Published:** 2025-05-06

**Authors:** Stanislav Bureš, Petr Hekera, Paulína Jašková

**Affiliations:** 1 Institute of High Mountain Biology, University of Žilina, Tatranská Javorina 7, 059 56 Tatranská Javorina, Slovak Republic; 2 Department of Ecology and Environmental Sciences, Faculty of Science, Palacky University, Šlechtitelů 27, 783 71 Olomouc, Czech Republic; 3 Department of Mathematical Analysis and Applied Mathematics, Faculty of Science, Palacky University, 17 Listopadu 12, 771 46 Olomouc, Czech Republic

**Keywords:** Recovery, Lead, Calcium, Cadmium, Aluminum, Passerines, Invertebrates, Soil chemistry, Upland

## Abstract

The Eastern Sudetes Mountains (Northern Moravia, Czech Republic) were among the most polluted regions of Europe due to toxic metal depositions and acid rain, until the desulfurization of emissions from coal power stations and reduction of heavy industry which took place in the 1990s. This study provides a comparison of data on the levels of calcium, lead, cadmium, and aluminum in soil, meadow pipit (*Anthus pratensis*) nestlings, and their available diet in 1994–1999 and in 2017–2019. The soil pH and exchangeable amount of calcium and lead increased slightly. The concentration of calcium in potential prey (invertebrates) increased slightly, and lead and cadmium levels decreased. The amount of calcium and lead in nestling bodies decreased in 2017–2019, while cadmium and aluminum levels remained unchanged after accounting for nestling age. The age of nestlings had an effect on aluminum only, when its content decreased with age. The principal component analysis revealed close association between calcium and lead. The consequences of recent leaching of basic cations, mainly calcium, and increasing bioavailability of lead in soils for future reproduction of the meadow pipit are discussed.

## Introduction

Even though birds are considered good biomonitors of toxic metal load (Becker 2003; Furness 1993; Janiga 2001; Nyholm 1995; Scheuhammer 1987; Williams et al. 2017) and mountain ecosystems are heavily polluted and vulnerable in general, both due to long-range transport of pollutants and as a legacy of mining activities in the past (Janiga 2008; Le Roux et al. 2020; Shotyk et al. 2002), little attention has been paid to toxic metal contamination of passerines breeding in the mountains, with the exception of alpine accentors (*Prunella collaris*) (Janiga 2002; Janiga and Haas 2019).

Despite well-established knowledge of long distance emission transfer (Ackermann and Hanrahan 1999; Hédl et al. 2011; Reiners et al. 1975; Rühling and Tyler 2004; Shotyk and Le Roux 2005; Steinnes and Lierhagen 2018), high depositions in alpine areas, often increasing with altitude (Hédl et al. 2011; Janiga 2008; Shotyk et al. 2002; Zechmeister 1995), decrease in abundance of mountain bird species in alpine meadows (Bureš et al. 2000; Flousek et al. 2015; Lehikoinen et al. 2019) and assumed negative effects of toxic metals especially during increased metabolism of fast growing nestlings (Belskii and Belskaya 2021; Dauwe et al. 2004; Ding et al. 2020; Eeva and Lehikoinen 1996; Nyholm 1995, 1998; Pinowski et al. 1993; Saulnier et al. 2020; Scheuhammer 1987; White et al. 2022), and embryonic development (Eeva and Lehikoinen 1995; Orlowski et al. 2016) have not received sufficient attention.

The reduction of leaded petrol use in 2021 marked the end of leaded petrol worldwide, after it had contaminated air, dust, soil, drinking water, and food crops for the better part of a century especially between 1960 and 1980 (Hagner 2002). Improvement in technologies during the 1990 s leading to reduced industrial emissions has created conditions for the recovery of ecosystems in formerly negatively affected areas. In birds, the results differ among published studies. Some have reported rapid improvement in reproductive parameters (Berglund et al. 2012; Eeva and Lehikoinen 2000, 2015) while others show slow recovery in these variables (Belskii and Lyakhov 2022; Berglund and Nyholm 2011). However, there are a large number of environmental factors which affect the impact of industrial pollution on bird populations. Apart from altitude, weather, and soil conditions, the sensitivity to toxic heavy metal load is species-specific, due to different foraging strategies, physiology, and behavior (Barton et al. 2023; Berglund et al. 2011; Eeva and Lehikoinen 1996).

There is evidence from studies on the influence of pollution and recovery of passerine populations’ geographical bias (Barton et al. 2023), as the majority of studies were carried out in the northern hemisphere, mostly in northern Europe, and close to regionally important sources of emissions. To the best of our knowledge, there has been no investigation that has compared concentrations of toxic metals in nestlings of a mountain passerine species over the years, from an area where the quantity of depositions decreased.

The depositions of industrial dust (often rich in calcium), acid rains, and toxic metals were very high in Central Europe after the second world war until the end of the 1990 s (Grafická ročenka 2017; Hůnová 2020; Kopáček and Veselý 2005; Moldan and Schnoor 1992). Acid rains caused destruction of large areas of spruce forests and contributed to changes in vegetation in the alpine meadows (Rotter and Purchart 2022). Apart from the deleterious impact of leaded petrol, the main sources of acid rains and toxic metals in Central Europe were the industrial and mining areas in the so-called Black Triangle on the Polish-German-Czech borders (Mc Neil 2000), coal power stations, and large mining and industrial areas from both sides of the Sudetes Mountains along the Czech-Polish border to the east as far as Ostrava (Czech Republic) and Katowice (Poland) industrial aglomerations (Rotter and Purchart 2022). Due to prevailing north-west winds, the mountains were heavily polluted up to the Tatra Mountains in the Slovak Republic. The level of emissions diminished by degrees during the last years of the twentieth century as a result of desulfurization of emissions from coal power stations and reduction of heavy industry (Grafická ročenka 2017; Hůnová 2020).

The chronic dietary exposure of bird species to heavy metals causes various symptoms. Poisoning has both physiological and behavioral impacts, and thus, it can negatively affect both general health and reproduction, mainly during the period of rapid growth in nestlings of passerines, which are extremely sensitive to intoxication (Barton et al. 2023; and see literature cited above). The negative impact of lead and cadmium is due to, among other factors, acidification of the environment which contributes to increasing the bioavailability of heavy metals and reduced calcium availability from soils to food chains (Scheuhammer 1991). The acidification also causes a decline in snail abundance which is an important additional source of calcium for creating eggshell and the skeletal growth of nestlings (Bureš and Weidinger 2000; Graveland et al. 1994). Moreover, as the chemical properties of the lead and cadmium are similar to calcium, the Ca^2+^ binding proteins, as parathyroid hormone (PTH), created by the parathyroid glands, which regulates the body’s blood level of calcium and phosphorus, and calmodulin (CaM), an intracellular trigger protein reported to be a molecular target for Pb^2+^ binding, readily bind to heavy metals, especially if the calcium levels are low (Fullmer 1992; Fullmer et al. 1985; Kirberger et al. 2013). For these reasons, it is sometimes difficult to determine whether poor breeding performance in birds is caused by calcium deficiency or heavy metal toxicity (Graveland 1998).

The aim of this study was to compare the content of non-essential metals (cadmium, lead, and aluminum) and calcium in the meadow pipit (*Anthus pratensis*) nestlings at the end of the period of depositions of industrial pollution in the 1990 s with samples taken 20 years later. As additional data to explain any assumed recovery of the mountain environment, samples of soil and potential diet of the meadow pipit were taken for chemical analyses in the two periods as well.

## Material and methods

### Study area and species

The study was carried out on the central ridge of the Jeseníky Mountains (Eastern Sudetes Mountains) around the summit of the Vysoká Hole and Petrovy kameny in the Czech Republic (50° 04′ N, 17° 15′ E, 1350–1464 m.a.s.l). Non-grazed grassy alpine meadows with small scattered rocks, stunted Norwegian spruce (*Picea excelsa*), and fields of blueberries (*Vaccinium myrtillus*) dominate the area. Prevailing bedrocks are metamorphic siliceous rocks—phyllites and quartzites with low content of basic cations. Soil types are strongly leached acidic shallow podzols with a high content of humus and very low content of basic cations, mainly calcium.

The meadow pipit is a small (16 g), prevailing insectivore passerine (Bureš 1994; Bureš and Weidinger 2000), which builds its nest in grass and blueberries and normally raises 4–5 nestlings, which are fledged at 13 days old (own data). The abundance of the pipits, mainly in the flagship species for the Eastern Sudetes Mountains, the water pipit (*Anthus spinoletta*), decreased rapidly during the 1990 s. One possible cause was intoxication, and another, insufficient calcium availability. We carried out the following.

The pipit nests were found mainly during incubation and checked regularly once every 2–3 days. Dead nestlings (one per nest), which had died usually after heavy rains or snowstorms, were taken, the digestive tract was removed, and nestlings were frozen and lyophilized before chemical analyses. Sixty-six dead nestlings were sampled during the first period (1994–1999) and an additional 10 nestlings during the second period (2017–2019). The dead animals are usually used for similar comparative studies (Beyer et al. 1988; Janiga and Janiga 2023; Pinowski et al. 1993), mainly for ethical and legal reasons. The first study, 1994–1999, was immediately after a period of heavy pollution depositions and acid rains (Kopáček and Veselý 2005).

The four soil samples, about 200 g of soil from the upper 5 cm layer of soil without litter, were taken from randomly chosen spots in the study area during the meadow pipit nestling period in 1996 (Bureš and Weidinger 2000) and in 2019. The places were marked by a plastic tube for sampling in future. The samples were placed in a metal-free plastic bag, immediately taken to the laboratory, dried, and analyzed.

The potential prey was sampled by a standardized method of sweep nets (Frey-Roos et al. 1995) along four transects located close to the place of soil sampling (each time by 100 sweeps) when meadow pipits fed their first brood in 1997 (Bureš and Weidinger 2000) and in 2019. Sampled arthropods were then placed in plastic bags, transferred to a laboratory where only potential prey for meadow pipit was identified (pieces of vegetation and arthropods not recorded in meadow pipit diet by taxa or size were excluded) and dried to constant mass at 55 °C (details in Bureš et al. 1999, Bureš and Weidinger 2000). The summed samples of arthropods from both periods were analyzed by comparable chemical methods, except for the use of new analytical devices in samples from 2017 to 2019 (see below).

### Chemical analyses

The soil samples from both periods were extracted with deionized H_2_O to determine the active pH and with 1 M KCl solution to estimate the exchangeable pH (ISO/DIS 10390) (Zbíral 1995). The pH was measured in the extract with a glass electrode.

In the first period (1994–1999), dried and homogenized soil samples were mineralized in a microwave digestion unit Uniclever II (Plazmatronica, Poland), and the total calcium and lead content was determined after oxidation with H_2_SO_4_ and H_2_O_2._ The exchangeable calcium and lead fraction was measured after extraction with 1 M ammonium acetate at pH = 7, using an AAS Avanta ∑ (GBC Australia).

In the later period (2017–2019), dried and homogenized soil samples were mineralized in a microwave decomposition device SpeedWave SW2 (Berghof company) in a mineralization mixture of HNO_3_ and HCl (Berghof 2010). The mineralizates were analyzed on the SAvanta AAS (GBC Australia). Cadmium and lead were analyzed in acetylene-air flame and Ca in acetylene-nitrious oxide flame.

Determination of the exchangeable content of calcium, lead, and cadmium: dried and homogenized soil samples were leached using the Mehlich III extraction agent (EDTA, CH_3_COOH, HNO_3_) (Zbíral 1995). The same device was used for AAS analysis of calcium, lead, and cadmium in leachates.

Assessment of calcium, aluminum, lead, and cadmium levels in samples of potential prey and dead nestlings from 1994 to 1999 were done at the Ekocentrum Ostrava, a laboratory accredited by the Veterinary Administration of the Czech Republic, by plasma emission spectrophotometry (OES-ICP, AES-ICP) on a JOBIN–YVON JY 138 + in these wave lengths: calcium 315.887 nm, cadmium 228.802 nm, lead 220.353 nm, aluminum 308.215 nm.

The samples of potential prey and dead nestlings from 2017 to 2019 were mineralized in the Berghof SpeedWave SW2 microwave decomposition device in HNO_3_, H_2_O_2_ mineralization mixture (Berghof 2010). The concentrations of metals of interest (except for calcium) were determined by inductively coupled plasma mass spectrometry (ICP-MS, Agilent 7700 x, Agilent, Japan) after microwave digestion with HNO_3_ and HCl. The ICP-MS spectrometer was operated in [He] mode to overcome potential spectral interferences. Calcium concentration was determined using an atomic absorption spectrometer (AAS, SAvanta, GBC, Australia) with an acetylene-air flame and LaCl_3_ as a releasing agent. The external calibration using certified reference materials – water calibration solutions of each metal (Analytika Ltd., Czech Republic) was implemented to obtain concertation values for both ICP-MS and AAS.

### Statistical analysis

We analyzed the concentrations of various chemical elements from two periods (1993–1999 and 2017–2019), where sample sizes differed between periods. To account for these unequal sample sizes and to assess potential confounding due to nestling age, we employed an analysis of covariance (ANCOVA) using Type III sums of squares, which is recommended for unbalanced designs. The purpose of ANCOVA is to compare the mean values of a dependent variable across several groups while controlling for one or more covariates. Specifically, we modelled each element as the dependent variable, with the main categorical factor (period) as the independent variable and nestling age as a covariate. This approach allowed us to test the overall significance of the period factor after adjusting for nestling age. Subsequently, we fitted a linear model to the data, which enabled a more detailed estimation of the individual coefficients—specifically quantifying how each predictor (period and age) influenced the measured element concentrations. All analyses were conducted in R software (version 4.4.0), with statistical significance set at a 5% level (*α* = 0.05). Model assumptions were evaluated by inspecting residual plots, and data transformations were explored if necessary.

**Fig. 1:**
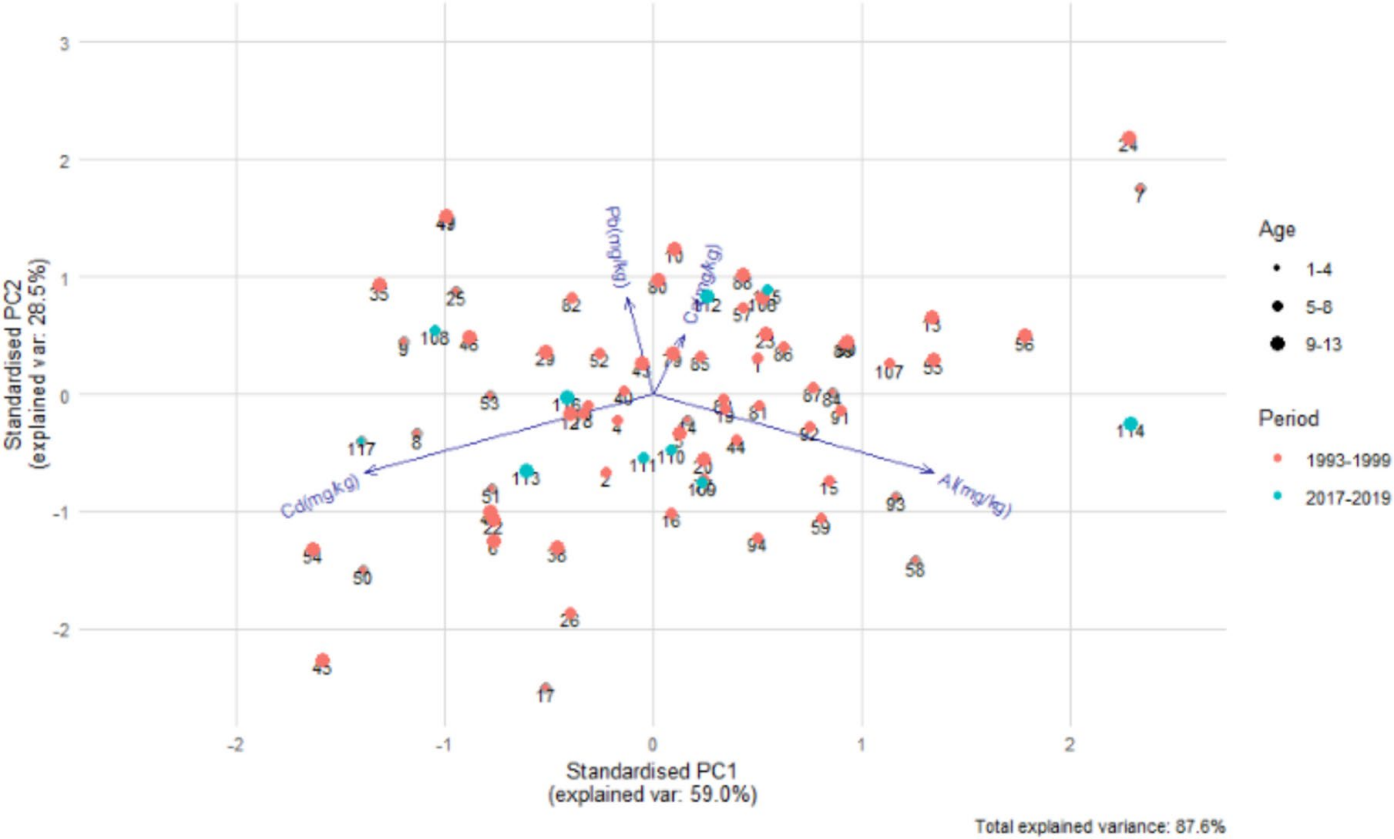
Compositional PCA biplot of studied elements between the two study periods (1994–1999 vs. 2017–2019). The x-axes and y-axes represent the first two principal components, which explain 87.6% of the total variance

Principal component analysis (PCA) was employed to identify the main sources of variability in the data. The data are represented through principal components, which are new variables constructed as linear combinations of the original variables. The results are illustrated in Fig. 1, which displays a biplot, a two-dimensional visualization of both objects and variables within a single graph. The x-axes and y-axes represent the first two principal components, capturing the largest proportion of variability in the dataset. In the plot, points represent individual objects (nestling), size of point represents age category, while arrows correspond to the original variables, indicating how they contribute to the principal components.

The lengths of the arrows are roughly proportional to the variances of the statistical features and highlight which variables exert the most influence on the arrangement of the observations in the graph. The direction of the arrows aids in interpreting the significance of individual features in the data. The cosine of the angle between two arrows reflects the correlation coefficient between the respective variables: the smaller the angle, the stronger the linear relationship between the corresponding variables.

## Results

During the first study, 1994–1999, after a period of serious acidification and high depositions of toxic metals, the soil was slightly more acidic (Table 1) than during the second period (2017–2019). The total amount of calcium in the soil decreased, but the exchangeable amount of calcium increased slightly in 2017–2019 (Table 1). Both total and exchangeable content of lead in soil slightly increased in this period (Table 1).

**Table 1:** Comparison of soil pH and chemical elements in soil and potential prey between the two study periods (in 1994–1999 from Bureš and Weidinger (2000))

Soil	pH (KCl)	pH (H_2_O)	Ca total, mg/kg	Ca exch., mg/kg	Pb total, mg/kg	Pb exch., mg/kg	Prey	Ca total, mg/kg	Pb total, mg/kg	Cd total, mg/kg
1993–1999	2.80	4.69	2195	202.4	59.10	1.94	1993–1999	716	5.27	1.15
2017–2019	3.13	4.92	736.8	263.0	87.16	7.91	2017–2019	767	1.20	0.85

The concentrations of calcium increased slightly, and the amount of lead and cadmium decreased in potential prey till the second study period (Table 1). The taxonomic composition of potential prey from both periods is shown in Table 2.

**Table 2:** Composition of potential prey in compared periods (16 samples in 1997 and 8 samples in 2019). The taxa are sorted according to their abundance in 1997 (Bureš et al. 1999)

Taxon	1997		2019	
	*n*	%	*N*	%
Aphidoidea	1198	62.9	423	49.2
Cicadomorpha	384	20.2	182	21.2
Diptera	193	10.1	178	20.7
Hymenoptera	73	3.8	32	3.7
Araneida	29	1.5	17	2.0
Coleoptera	21	1.1	11	1.3
Lepidoptera	2	0.1	12	1.4
Others	6	0.3	4	0.5
Total	1906	100	859	100

The chemical analysis of nestling bodies revealed differences between the two periods. The compositional PCA biplot (Fig. 1) explains 87.6% of the total variance. The PCA analysis (Fig. 1) revealed a positive correlation between calcium and lead (similar direction and small angle between the elements), a negative correlation between cadmium and aluminum, and the weakest correlation between cadmium and either lead or calcium.

The predictions from PCA analysis were accurated by ANCOVA analysis and linear regression models, which showed similar content of aluminum (*t* = − 1.843, *P* = 0.069) and cadmium (*t* = 1.523, *P* = 0.221) and decreased content of calcium (*t* = − 2.703, *P* = 0.008) and lead (*t* = − 2.942, *P* = 0.004) in nestling bodies during the two decades, if the contents were checked for the age of nestlings. There was no effect of nestling age on mineral content, except for aluminum which decreased with age (*t* = − 2.321, *P* = 0.023).

**Fig. 2:**
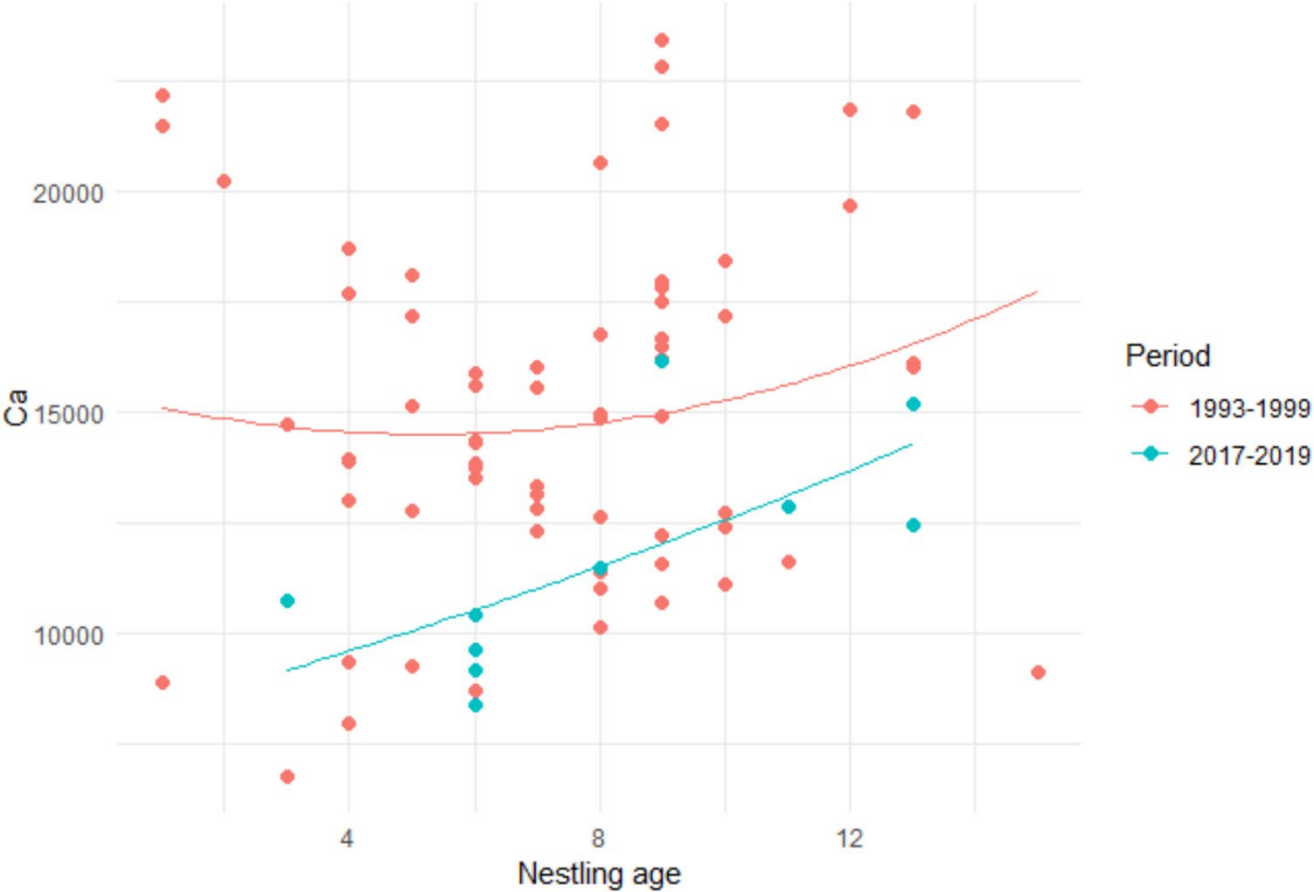
Calcium content in nestlings of various ages in the two study periods (mg/kg)

**Fig. 3:**
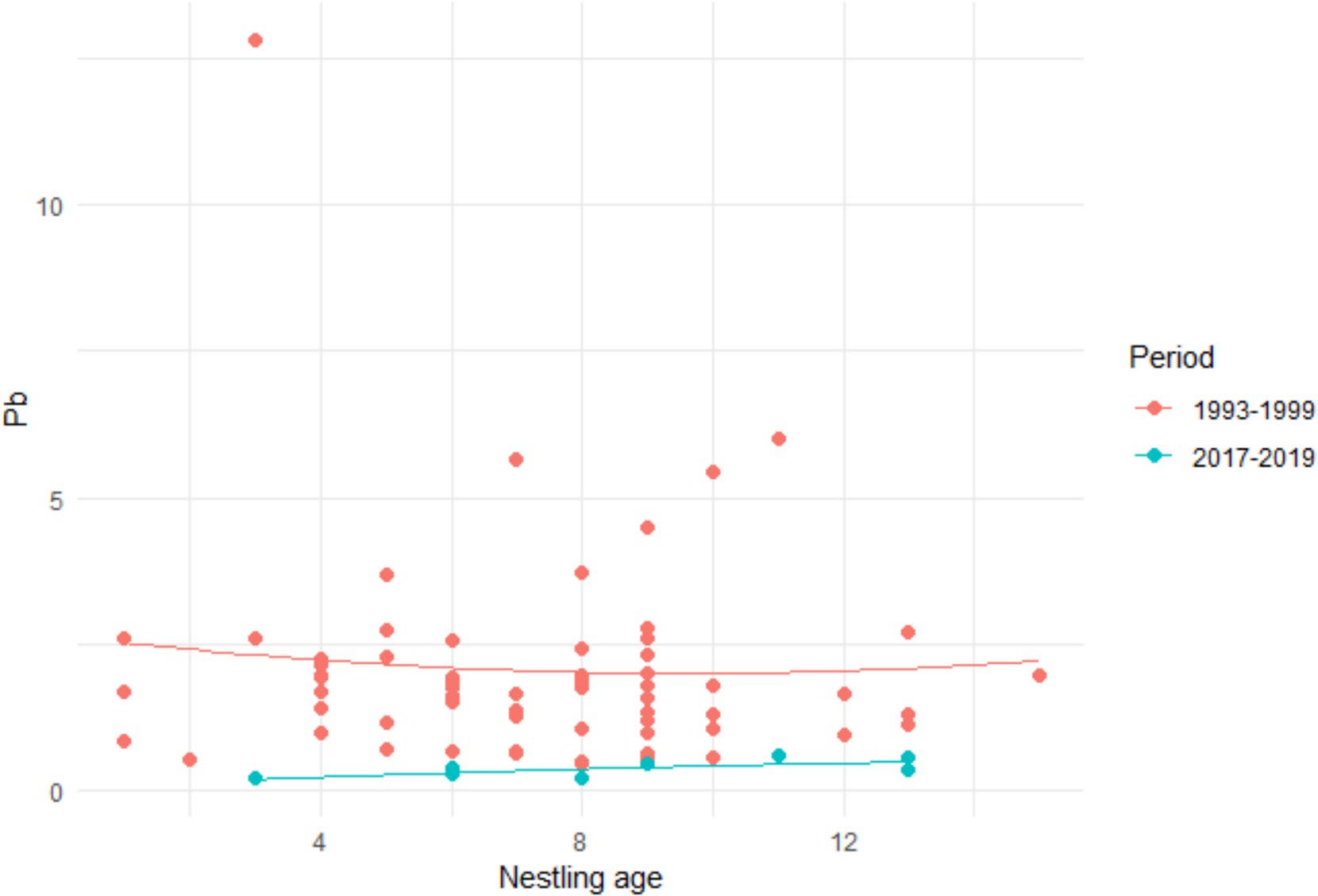
Lead content in nestlings of various ages in the two study periods (mg/kg)

The differences in calcium and lead content in nestlings of various ages between the compared periods are illustrated in Fig. 2 and Fig. 3. The fitted quadratic polynomial curves illustrate the trend of calcium or lead concentration with increasing age and provide a basis for comparative studies.

Thus, the results showed a discrepancy between the content of the chemical elements in soil and in potential prey and nestling bodies, which will be discussed.

## Discussion

The data confirm a predicted but weak decline in heavy metal contamination of pipits in the alpine meadow of the Eastern Sudetes Mountains from 1994–1999 to 2017–2019. Lead and cadmium content in prey slightly decreased, while soil pH and calcium content in prey increased mildly, and calcium and lead content in nestling bodies decreased during the two periods. However, exchangeable content of lead in soil increased, in calcium slightly as well, but the total content of calcium seriously declined.

Hruška et al. (2023) confirmed that upper mineral soils are still acidified in the “Black Triangle” of Central Europe, despite decreased SO_2_ emissions, increased precipitation pH, and that it is not soil acidification, but air pollution that has more influence on forest health. Hédl et al. (2011) confirmed rapid soil acidification due to pollution between 1941 and 2003 in the upper part of the Eastern Sudetes Mountains and their data are comparable with the years of 1993–1999 in this study and this despite the fact that the concentrations of SO_2_ here were much lower than in the Black Triangle and were up to 20 μg/m^3^. Lead is more soluble in an acidic environment. It accumulates mainly in the humus layer of soils; its downward migration velocity is very slow (0.3–8 mm/year), faster in acidic soils, and recovery of soils can take hundreds of years (Maskall et al. 1995; Shotyk and Le Roux 2005; Tyler 1978; Vorobeichik and Kaigorodova 2017). However, the total lead soil concentration is not a good indicator of the lead availability to plants, as lead extractable with diluted acids only is considered plant available (Hagner 2002; Vanmechelen et al. 1997). Surprisingly, the content of both total and exchangeable lead in soil increased from 2017 to 2019, probably because of slower leaching from humus and top soil layers when the pH slightly increased (Table 1). However, the concentrations of lead are far below the values for critical lead soil contamination (150 mg/kg) (Tyler 1992). In contrast, total calcium content in soil decreased markedly, and exchangeable calcium level slightly increased, but remained very low, under estimated minimal limit of about 500 mg/kg. This might be caused by fast electrostatic removal of dust emissions rich in base cations (mainly calcium) from the 1980 s until the late 1990 s which was earlier than SO_2_ removal. Thus, the ratio of SO_2_ to dust peaked late in the 1990 s (Hruška et al. 2023). Dust rich in cations could neutralize precipitation acidity and add calcium in soil. Later, high storage of sulfur depositions in soil in the area (Mörth et al 2005; Novák et al. 2000), which limits recovery of soils, and low storage of calcium in oligobasic soils could cause gradual leaching of calcium, and to increased concentrations of bioavailable lead.

Leaching of lead, which is strongly bound in the humus layer, can be very slow, mainly when the pH slightly increases in the area. But calcium leaching can progress as its solubility is faster and the soil is still acidic enough. It seems that binding mechanisms can be disrupted in the sorption complex of the soils here, especially when high nitrate deposition (10–15 kg/ha/year) (Oulehle et al. 2016) is higher than the estimated limit (5–10 kg/ha/year) for such ecosystems (Bobbink and Hettelingh 2010). The latter leads to soil acidification and limits the availability of calcium and other essential elements (Scheuhammer 1991). Moreover, this contributes to the reduction of plant species of alpine meadows in the area and to the rapid spread of grass and bilberries, which create an acid humus layer, and the negative processes are recently supported by warming (Chapin et al. 1995).

The composition of the potential prey was similar in both periods except for higher abundance of Aphidoidea in 1997 and Diptera in 2019. It can be explained by the rise in the number of Aphidoidea in areas influenced by air pollution (Holopainen et al. 1991) and ephemeral emerging of Diptera (mainly family Bibionidae) in 2019. The taxa have similar content of calcium (Studier and Sevick 1992). A number of factors (pH, organic matter, clay, cation exchange capacity) influence the bioavailability of metals to soil invertebrates. The uptake and elimination of cadmium and lead in invertebrates is species-dependent and without any clear phylogenetically related trend due to various mechanisms of detoxication (Ardestani et al. 2014). It is known that lead and cadmium concentrations decline with increasing trophic levels, e.g., plant–invertebrates (Dauwe et al. 2004; Zhuang et al. 2009), mainly owing to storage of the metal in excreta. Unfortunately, there is a paucity of data on toxic heavy metal uptake and toxicokinetics in larvae of Bibionidae, Sciridae, and Tipulidae, which are, as imagoes, along with Aphids and Lepidoptera caterpillars, the main diet of the meadow pipit nestlings (Bureš 1994; Bureš and Weidinger 2000), and they are not known in the mountain alpine environment. As the pH of soil slightly increased and depositions of cadmium and lead decreased in the Czech Republic between 1994 and 2017, increased availability of calcium and decreased content of cadmium and lead in potential prey were expected. However, the content of calcium increased negligibly and was at the lowest levels known from the literature (Graveland and Van Gijzen 1994; Studier and Sevick 1992). In contrast, lead and cadmium concentrations were in ranges known from heavily polluted areas (Dauwe et al. 2004; Roth 1993; Zhuang et al. 2009).

Dead nestlings were used for chemical analysis because of ethical and legal reasons. Dead animals have been usually used for similar analysis (Beyer et al. 1988; Janiga and Janiga 2023; Pinowski et al. 1993). The samples can be considered representative, because the same method of sampling (one dead nestling per nest) was used in both periods. Moreover, there was a last chance for this kind of study, because the abundance of the meadow pipit declined dramatically in recent years. Thus, a low number of samples in the second period can be acknowledged especially when appropriate statistical methods were used.

The concentration of calcium in nestling bodies in 1994–1999 was similar and appears to have a similar trend as slightly increased concentration before fledging, as Hagen et al. (1976) found in meadow pipit nestlings in south Norway. However, the concentration was lower in 2017–2019. The polynomial function (Fig. 2) enables the identification of non-linear relationships that might be influenced by environmental or physiological factors. Comparison with results of other authors who have published the calcium levels in passerines is not possible as these studies did not analyze whole body, or they did analyze breeding females during the egg-laying period (Krementz and Ankney 1995; Pinowska and Krasnicki 1985).

The parents of the meadow pipit were forced to invest more energy and time searching for aditional calcium sources (snailshells, rodent bones, etc.) during reproduction, as Graveland and Berends (1997) showed in aviary experiments in the Great Tit (*Parus major*) and, thus, compensate for calcium deficiency in the area in 1994–1999 (Bureš and Weidinger 2000, 2001). Snails were very rare in the area. The Genus *Semilimax*, which does not create a full snail shell (adaptation to calcium deficiency), prevailed (Bureš and Pokorná 1996). Bureš and Weidinger (2000) showed that arthropods covered only 16% of the total calcium requirements for the meadow pipit nestling growth and snail shells eaten about 148%, despite paucity of snails in the area. Thus, slightly increased calcium content in potential prey in 2017–2019 plays probably a negligible role in calcium intake for nesling growth, and additional calcium sources (mainly snail shells) play essential role as in 1994–1999.

The importance of sufficient dietary calcium intake in reducing the accumulation of lead and cadmium both in altricial and precocial bird species has been confirmed in a number of studies (Berg et al. 1980; Dauwe et al. 2006; Eeva and Lehikoinen 2004; Fullmer 1997; Saulnier et al. 2020; Scheuhammer 1996; Six and Goyer 1970; Snoeijs et al. 2005). Although no symptomps of intoxication, or calcium deficiency have been recorded, the content of calcium in nestling bodies decreased. The concentrations of lead decreased as well, probably because of decreased content of lead in potential prey, and are below the estimated danger limit (100 mg/kg d.w.) for nestlings of passerines (Scheuhammer 1991). But, recent studies have confirmed the negative impact of sublethal concentrations of lead on the behavior of passerines (Barton et al. 2023; Di Liberto et al. 2024). Unfortunately, we do not know the current abundance of snails in the area, and there is lack of studies on how lead and cadmium influence the PTH and calcitonin levels which influence retention or excretion rates of calcium (Babič Leko et al. 2022).

The decrease in cadmium content in potential prey is low during the study periods. Random higher cadmium intake in nestlings in the past could have been caused by discarded Ni–Cd batteries, which were found close to the walking tours on several occasions. Seven out of 9 nests (sampled nestlings), where cadmium concentration was more than 1 mg/kg d.w., were at a distance of less than 40 m from the walking tour. No such high concentration was recorded recently. The white content of the battery resembles a calcium source and could be consumed as shown in experiments reported by Bureš and Weidinger (2001). The production of these batteries was prohibited in the EU in 2008, and old batteries were overgrown by grass from that time. The PCA analysis supports the idea (see above).

Despite the somewhat optimistic results, as concentrations of lead and cadmium decreased and calcium concentration remained similar in potential prey, the decreased concentration of calcium in nestling bodies indicates insufficient availability of additional calcium sources in the area. Moreover, the diminished levels of total soil calcium are a warning signal, especially when sulfur depositions in soils and recent nitrate depositions are high. Thus, the data predict difficult recovery of formerly heavy polluted mountain areas with naturally oligobasic soils. The calcium availability can be a limiting factor for snail reproduction, and thus birds as well in the near future, and this can influence both the direct reproduction of these animals and the negative effects of increased bioavailability of toxic metals.

## Data Availability

Data matrices will be provided by the corresponding author on reasonable request.
